# The Evaluation of the Adjunctive Therapeutic Value of Mindfulness-Based Stress Reduction Therapy for Patients With Nasopharyngeal Carcinoma Induced Moderate Depression

**DOI:** 10.62641/aep.v53i3.1749

**Published:** 2025-05-05

**Authors:** Zeyu Zheng, Jing Han

**Affiliations:** ^1^Department of Otolaryngology, Head and Neck Surgery, The Second Affiliated Hospital of Harbin Medical University, 150000 Harbin, Heilongjiang, China

**Keywords:** adjunctive therapeutic, Mindfulness-Based Stress Reduction Therapy, nasopharyngeal carcinoma, moderate depression

## Abstract

**Background:**

Nasopharyngeal carcinoma (NPC) presents a substantial challenge for patients, impacting their physical and psychological well-being. Patients may experience moderate depression, anxiety, and reduced quality of life due to the disease and its treatments. Therefore, we retrospectively analyzed the adjunctive therapeutic potential of mindfulness-based stress reduction (MBSR) therapy for NPC patients with moderate depression.

**Methods:**

Psychological parameters were assessed using standardized scales, including the Hamilton Depression Scale-17 (HAMD-17), Self-Rating Depression Scale (SDS), Perceived Social Support Scale (PSSS), the Short-From-12 Health Survey (SF-12), and Mindfulness Attention Awareness Scale (MAAS). Statistical analyses were performed to compare the two groups.

**Results:**

A total of 131 patients including 67 patients with control group and 64 patients with Mindfulness-Based Stress Reduction therapy group were included. After 8 weeks of treatment, the MBSR therapy group showed significant improvements in psychological parameters, including depression, anxiety, perceived stress, quality of life, and mindfulness attention awareness (*p* < 0.05), compared to the control group. Additionally, the MBSR therapy group reported significantly higher overall satisfaction with treatment, willingness to recommend treatment, and perceived benefit from treatment (*p* < 0.05).

**Conclusion:**

The study findings support the adjunctive therapeutic value of MBSR therapy in improving psychological outcomes and patient satisfaction among individuals with NPC-induced moderate depression.

## Introduction

Nasopharyngeal carcinoma (NPC) presents a complex and multifaceted challenge, affecting patients not only physically but also psychologically [1]. The management of NPC generally involves aggressive treatment modalities, including radiation therapy and chemotherapy, which can significantly influence patients’ psychological well-being [2, 3]. The prevalence of moderate depression among NPC patients underscores the critical need for holistic care to address both physical and psychological aspects [4, 5]. Consequently, there is a growing interest in exploring adjunctive therapeutic interventions to improve the psychological well-being of patients with NPC-induced moderate depression.

Depression, anxiety, and reduced quality of life are among the common psychological manifestations encountered by patients with NPC [6, 7]. The interplay of disease-related stress, treatment-related side effects, and the chronic nature of NPC can contribute to the development or exacerbation of depressive symptoms [8, 9]. The psychological toll of managing NPC is further compounded by uncertainty, fear, and the overall emotional burden associated with the disease, underscoring the need to explore therapeutic approaches that can help mitigate these psychological distresses.

Mindfulness-based stress reduction (MBSR) therapy has emerged as a promising adjunctive intervention for enhancing the psychological well-being of patients with various medical conditions, including cancer [10]. Rooted in the principles of mindfulness, MBSR provides a structured, evidence-based approach to improving present-focused awareness, non-judgmental thinking, and adaptive coping strategies [11]. Its application in cancer care has gained attention due to its potential to enhance emotional regulation, improve quality of life, and alleviate psychological distress [12]. Studies across diverse cancer populations have shown promising results, indicating that MBSR therapy can positively impact the psychological well-being of patients [13, 14].

From this perspective, evaluating the adjunctive therapeutic potential of MBSR therapy for patients with NPC-induced moderate depression is a compelling area of investigation. While existing literature has provided insights into the benefits of mindfulness-based interventions within the broader scope of cancer care, targeted research focusing on the specific needs and challenges faced by patients with NPC-induced moderate depression is required. Therefore, this study aimed to comprehensively assess the adjunctive therapeutic value of MBSR therapy, focusing on the unique psychological needs and challenges faced by patients with NPC.

## Materials and Methods

### Study Design

This retrospective cohort study included NPC patients with moderate depression admitted to the Second Affiliated Hospital of Harbin Medical University, China, between January 2023 and June 2023. This study was approved by the ethics committee of the Second Affiliated Hospital of Harbin Medical University, China (Approval No.: KS20240101). The study design adhered to the Declaration of Helsinki [15], and informed consent was obtained from each study participant.

Patients were divided into two groups based on different treatment modalities: a conventional treatment group (n = 67) and an MBSR therapy group (n = 64). The patient selection process involved careful consideration and respect for patient preferences. Initially, physicians provided information about available treatment options, outlining their benefits and potential risks in a clear and understandable manner to ensure patient’s comprehensive understanding. Subsequently, shared decision-making occurred between patients and physicians through open and honest discussions regarding available treatment options, considering the patient’s medical condition, personal values, and preferences. Importantly, all decisions were made within the framework of medical ethics, ensuring the effective preservation of patients’ autonomy and their right to information throughout the decision-making process.

### Eligibility Criteria of Study Participants

Inclusion criteria included patients with pathologically confirmed NPC, aged over 18 years, with disease duration of ≥6 months and normal cognitive function, who can understand and participate in the questionnaire survey. Moreover, only the patients with complete medical records or information were included in the study cohort. During hospitalization, patients underwent depression assessment using the Hamilton Depression Scale (HAMD)-17 in conjunction with the criteria from the Diagnostic and Statistical Manual of Mental Disorders, fifth edition, (DSM-5) [16]. In HAMD-17 score, 17–24 points indicate moderate level of depression symptoms.

Furthermore, exclusion criteria were set as follows: Patients with other severe chronic diseases affecting vital organs such as the heart, liver, or kidney, those with a pre-existing diagnosis of depression and receiving related interventions at the time of consultation or before admission, or patients with other psychiatric disorders, patients with secondary primary cancer or distant metastasis, those with concurrent severe physical illnesses, and those simultaneously using other medications or non-pharmacological treatments during therapy.

### Treatment Approach 

Patients in the control group received conventional nursing care as follows:

(1) A care plan was developed based on the specific circumstances of each patient. Basic knowledge about NPC was explained to patients and their families, including details about etiology, treatment methods, and adverse reactions during treatment.

(2) To maintain oral hygiene, patients were directed to brush their teeth after meals and to stay hydrated by drinking about 2500 mL of water daily. Additionally, patients were advised to consume more high-protein, high-calorie, easily digestible food and quit smoking and drinking.

(3) For patients with epistaxis, nursing staff should assess the comfort of patients and promptly inform the physician. In such cases, patients were guided to lie down, turn their heads to one side in time to remove the blood from their mouth and nose and keep the respiratory tract unobstructed. Furthermore, their vital signs were closely observed following the physician’s instructions.

(4) Patients undergoing radiotherapy were advised to keep their skin clean and dry and wear cotton underwear and collarless clothing to reduce neck skin irritation. They were directed to wash irradiated skin gently using a soft towel. If skin damage occurs, such as itching, dryness, darkening, desquamation, or blisters, they were instructed to follow the physician’s advice for treatment and regularly observe the skin for any changes. Additionally, patients were asked to drink more water during radiotherapy to increase urine output, accelerate toxin excretion, and reduce nephrotoxicity. After radiotherapy, patients were guided to perform mouth-opening training to prevent sub-temporal joint dysfunction.

(5) Enhancement monitoring was performed by communicating with patients; their confidence and sense of security were substantially improved. Meanwhile, nursing staff closely observed patients’ ideological dynamics for early psychological assessments and timely psychological and behavioral interventions to prevent accidents.

The MBSR treatment group received conventional nursing and MBSR treatment. The conventional nursing care was the same as that of the control group. The MBSR treatment included the following:

(1) A mindfulness-based stress intervention team was established, comprising five MBSR-certified clinical therapists and three nurses who had received mindfulness training.

(2) On the first day of admission, the team members explained the significance of MBSR treatment to the patients and their families. Moreover, they established a WeChat group to share activity videos with them, providing comprehensive implementation methods.

(3) An 8-week learning course was conducted, and MBSR learning was performed once a week. Patients were guided to carry out MBSR exercises according to the weekly learning contents in the rest of the time. The theme and content of the weekly learning course are shown in Table 1.

**Table 1.** **Weekly learning contents for MBSR treatment**.

Week	Learning contents	Learning time	Requirements
1	Patients were introduced to the knowledge of MBSR and the eight mindfulness attitudes: self-care, self-trust, natural development, calmness and peace, effortlessness, purity of heart, non-judgment, and positive affirmation. Patients were encouraged to carefully observe the smell, color, nature, and appearance of food, fully experience the process of chewing and swallowing food, and apply these eight attitudes to their daily lives.	30 minutes	Patients needed a 5-minute mindfulness eating practice each day.
2	The patients were guided to share their experience of mindfulness practice for 5 minutes. Patients were instructed to carefully observe the external environment, such as light, sound, and smell. They were guided to imagine the feeling of walking for the first time in their lives, deeply focusing on the contact between their body and the ground and the movement of their body during walking.	30 minutes	Patients were asked to include a 10-minute walk in their daily routine and maintain a 5-minute mindfulness eating practice every day.
3	The nursing staff spared 5 minutes for patients to share their experience with mindfulness eating and walking practices. After this, patients were guided through mindfulness breathing exercises. With gentle and soothing music playing in the background, patients were guided to relax their bodies, breathe slowly, and feel the sensation of inhaling and exhaling, as well as the fluctuations of their chest and abdomen.	30 minutes	Patients were asked to add 10-minute mindfulness breathing exercises to their daily routine, based on their practices in the first two weeks.
4	The nursing staff spared 10 minutes for patients’ communication, followed by a mindfulness meditation practice. In the gentle and soothing music, the nursing staff guided patients to observe their current emotions and thoughts through mindfulness breathing approaches.	30 minutes	Patients were guided to add 15-minute mindfulness meditation to their daily routines, based on their experiences in the first three weeks.
5	The nursing staff spared 10 minutes for patients’ communication. Once again, in the soft and soothing music, patients were guided to perform body scanning exercises, focusing on each part of the body, gradually from the left toe to the head. If the scanned part was found normal, patients were guided to relax it. If the scanned part encountered a feeling like pain, patients were guided to experience it until this feeling gradually subsided.	35 minutes	Patients were guided to select three activities from the previous four weeks and add 10 minutes of daily body scanning exercises.
6	The nursing staff spared 10 minutes for patients’ communication. Patients were encouraged to practice mindfulness yoga during soft and soothing music, which included 8 yoga movements. During this practice, they were guided to feel their emotions, thoughts, and feelings.	30 minutes	Patients were asked to choose 3 activities from the first five weeks, and add a 10-minute mindfulness yoga practice to their daily routines.
7	The nursing staff allocated 10 minutes for patients’ communication, followed by mindfulness emotion regulation. Patients engaged in mindfulness breathing and mindfulness meditation, applying the eight attitudes of mindfulness to their hospitalization.	30 minutes	Patients were asked to select 4 activities from the first six weeks of learning, and add a 10-minute mindfulness emotion regulation to their daily routines.
8	Patients were guided to discuss and summarize the changes brought by the mindfulness exercises.	20 minutes	Patients were encouraged to choose 4–5 activities from the first seven weeks of learning, ensuring a daily practice time of at least 45 minutes.

MBSR, mindfulness-based stress reduction.

### Data Collection 

#### Demographics and Clinical Parameters

Before treatment, patient demographic and clinical parameters were obtained through systematic case reviews. Demographic data included age, gender, body mass index (BMI), hypertension, diabetes, alcohol history, smoking history, duration of depression, marital status, education level, employment status, and Karnofsky Performance Score (KPS). Clinical parameters comprised tumor location, treatment method, tumor node metastasis (TNM) staging, and Eastern Cooperative Oncology Group (ECOG) performance status.

The KPS criteria were as follows: 100 points (normal), 90 points (normal activity with mild symptoms), 80 points (adequate activity with some symptoms), 70 points (self-care with limited activity), 60 points (occasional assistance, mostly self-care), 50 points (frequent care required), 40 points (unable to care for self, requiring assistance), 30 points (completely unable to care for self, requiring hospitalization), 20 points (very ill, requiring hospitalization), 10 points (moribund, close to death), and 0 points (dead).

The Cronbach’s α coefficient for the scale was 0.72 [17]. The Eastern Cooperative Oncology Group (ECOG) Performance Status was used to assess the physical status of the patients, with scores ranging from 0 to 5. A score of 5 indicates death, 4 indicates inability to perform self-care and being bedridden, 3 indicates partial self-care with more than 50% of daytime spent in a wheelchair or bed, 2 indicates self-care and ambulatory ability but no working capacity, with more than 50% of daytime spent in self-care activities, and 1 indicates ability to perform light work or walk freely, including office work and general housework, but not heavy labor. The Cronbach’s α coefficient for the scale was 0.73 [18].

#### Psychological Parameters

The psychological parameters of the two experimental groups were collected from the hospital medical record system before treatment, and after 8 weeks of treatment. These parameters included HAMD-17, Self-Rating Depression Scale (SDS), Perceived Social Support Scale (PSSS), the Short-From-12 Health Survey (SF-12), and the Mindfulness Attention Awareness Scale (MAAS).

Firstly, the HAMD-17 was used to assess the level of depressive symptoms in patients over the past 2 weeks. The scale consists of 17 items. A total score of <7 indicates no depressive symptoms, 7–16 indicates mild depressive symptoms, 17–24 indicates moderate depressive symptoms, and >24 represents severe depressive symptoms. The Cronbach’s α coefficient for the scale was 0.91 [19].

The SDS is a self-assessment questionnaire comprising 20 sub-items, with responses categorized into four levels. Each item is scored 1 to 4 based on the frequency that best represents one’s condition, with 1 indicating “never or rarely”, 2 representing “sometimes”, 3 indicating “often”, and 4 suggesting “always”. A higher score indicates more severe depressive symptoms. Furthermore, scores below 53 are considered normal, scores between 53 and 62 indicate mild depressive symptoms, 63 to 72 suggest moderate depressive symptoms, and 73 or higher indicate severe depressive symptoms. The reliability of this scale is reported as 0.73 [20].

The PSSS was utilized to determine perceived support from family, friends, and others. The scale consists of 12 items, each graded on a 7-point scale, ranging from 1 to 7, representing “very strongly disagree” to “very strongly agree”. A higher total score indicates a greater perceived level of social support. The Cronbach’s α coefficient for the scale was 0.907 [21].

The SF-12 scale comprises 12 items across 8 dimensions: physical functioning (PF), role limitations due to physical health (RP), bodily pain (BP), general health perceptions (GH), vitality (VT), social functioning (SF), role limitations due to emotional health (RE), and mental health (MH). It utilizes a percentage scoring method where a higher score indicates a higher quality of life. The Cronbach’s α coefficient for the scale was 0.89 [22].

The MAAS is a single-dimensional scale with 15 items to determine attention and awareness. The questionnaire employs a 6-point rating system, where 1 indicates “almost always” and 6 shows “almost never”. Higher scores indicate higher levels of mindfulness. The Cronbach’s α coefficient for the scale was 0.92 [23].

#### Treatment Satisfaction

After 8 weeks of treatment, patient treatment satisfaction was assessed using a self-designed questionnaire. The total score ranged from 0 to 10 and was categorized into four groups: very satisfied (8–10 points), satisfied (7–8 points), neutral (6–7 points), and dissatisfied (<6 points). Additionally, patient referral rates were also documented.

### Data Cleaning and Management

Prior to conducting data analysis, this study implemented a standardized data-cleaning process to identify and rectify any inconsistencies, errors, or missing values. This process included thorough examination of the dataset, removal of duplicate entries, correction of data input errors, and handling of missing values. Missing data were addressed using the Datawig and Pandas libraries in Python 3.6.0 (Python Software Foundation, Beaverton, OR, USA), utilizing deep neural networks. The proportion of missing data was kept below 5% to reduce potential selection bias, and sensitivity analysis was performed. Additionally, outcomes for cases lost to follow-up were calculated for both worst-case and best-case scenarios. If no significant differences were found in the conclusions, it was determined that loss to follow-up had minimal impact, ensuring the reliability of the conclusions. The final findings were presented after addressing the missing data.

### Post-hoc Analysis

Using G*Power 3.1.9.7, a post hoc analysis was performed based on the “Means: Difference between two independent means (two groups)” option for *t*-tests. The analysis was set to a two-tailed mode with an effect size (d) of 0.6 and an error probability (α) of 0.05. Subsequently, based on the sample sizes of the two groups, the Power (1-β error prob) was determined to be 0.926. However, it is crucial to interpret post hoc statistical power analysis with caution due to certain limitations that have been debated.

The post hoc statistical power analysis is highly sensitive to sample size and effect size, meaning that minor variations in these variables can lead to significant changes in the calculated power. This sensitivity may result in misinterpretation, making it challenging to draw definitive conclusions. Furthermore, post hoc statistical power analysis cannot establish causality. It does not determine whether the observed effect size results from sample size, variability, or the actual effect of the intervention or treatment. Therefore, interpreting post hoc statistical power as a conclusive measure of research quality or the significance of observed effects can be misleading.

### Statistical Analysis

The data were analyzed using SPSS 26.0 software (IBM, Armonk, NY, USA). Categorical data were expressed as [n (%)]. Given that the sample size was >40, the chi-square test was applied using the basic formula when the theoretical frequency (T) was ≥5, with the test statistic represented by χ^2^. However, when the theoretical frequency was 1 ≤ T < 5, the chi-square test was adjusted using a correction formula. For cases where the theoretical frequency was T <1, statistical analysis was conducted using Fisher’s exact probability method. The Shapiro-Wilk test was used to determine whether continuous variables followed a normal distribution. The continuous variables following a normal distribution were expressed as (mean ± standard deviation ($\bar{x}$ ± s)) and analyzed using the *t*-test. A two-sided *p*-value < 0.05 was considered statistically significant.

## Results

### Comparison of Demographic and Basic Data Between the Two Experimental Groups

A total of 131 patients including 67 patients with control group and 64 patients with MBSR therapy group were included. A comparison of pre-treatment characteristics between these two experimental groups showed no statistically significant differences in age, gender, BMI, hypertension, diabetes, alcohol drinking, smoking, duration of depression, marital status, education level, employment status, and Karnofsky performance status (*p* > 0.05, Table 2). These findings indicate comparable baseline characteristics between the two groups, supporting the validity of subsequent treatment comparisons.

**Table 2.** **Comparison of pre-treatment characteristics between the control and MBSR therapy groups**.

Parameters		Control group (n = 67)	MBSR therapy group (n = 64)	*t*/χ^2^	*p*-value
Age (years)		51.25 ± 6.32	50.87 ± 5.98	0.360	0.720
Gender (male/female)		38 (56.72%)/29 (43.28%)	35 (54.69%)/29 (45.31%)	0.055	0.815
BMI		24.15 ± 3.45	24.32 ± 2.14	–0.354	0.724
Hypertension		6 (8.96%)	7 (10.94%)	0.144	0.704
Diabetes		7 (10.45%)	5 (7.81%)	0.273	0.601
Alcohol drinking		34 (50.75%)	29 (45.31%)	0.387	0.534
Smoking		20 (29.85%)	22 (34.38%)	0.308	0.579
Duration of depression (months)		6.77 ± 1.89	6.92 ± 2.05	–0.438	0.662
Marital status					0.989*
	- Married	45 (67.16%)	43 (67.19%)		
	- Single	12 (17.91%)	11 (17.19%)		
	- Divorced	6 (8.96%)	5 (7.81%)		
	- Widowed	4 (5.97%)	5 (7.81%)		
Education level				0.293	0.864
	- High school	18 (26.87%)	15 (23.44%)		
	- College	42 (62.69%)	43 (67.19%)		
	- Master’s degree and above	7 (10.45%)	6 (9.38%)		
Employment status				0.024	0.988
	- Employed	50 (74.63%)	47 (73.44%)		
	- Unemployed	8 (11.94%)	8 (12.50%)		
	- Retired	9 (13.43%)	9 (14.06%)		
KPS		85.14 ± 3.76	85.59 ± 3.42	–0.709	0.480

* indicates Fisher test results. MBSR, mindfulness-based stress reduction; BMI, body mass index; KPS, Karnofsky Performance Score.

### Comparison of NPC Features Between the Two Experimental Groups

A comparison of NPC features between the two groups indicated no statistically significant differences in tumor group distribution, treatment group distribution, TNM stage (T) distribution, TNM stage (N) distribution, TNM stage (M) distribution, and baseline ECOG performance status distribution (*p* > 0.05, Table 3). These findings suggest a balanced representation of NPC features in both experimental groups, providing a robust foundation for evaluating the outcomes of MBSR therapy in patients with NPC-induced moderate depression.

**Table 3.** **A comparison of NPC features between the two experimental groups**.

Parameters		Control group (n = 67)	MBSR therapy group (n = 64)	χ ^2^	*p*-value
Tumor group				3.299	0.348
	- Oral cavity	11 (16.42%)	16 (25.00%)		
	- Oropharynx	23 (34.33%)	26 (40.62%)		
	- Larynx, hypopharynx	23 (34.33%)	16 (25.00%)		
	- Other	10 (14.92%)	6 (9.38%)		
Treatment group				0.797	0.939
	- Traditional radiotherapy	30 (44.78%)	30 (46.88%)		
	- External beam radiotherapy combined with brachytherapy	12 (17.91%)	14 (21.88%)		
	- Hypofractionated radiotherapy	12 (17.91%)	10 (15.62%)		
	- Radiotherapy coupled with chemotherapy	10 (14.92%)	7 (10.94%)		
	- Other	3 (4.48%)	3 (4.68%)		
TNM stage (T)				0.722	0.868
	- T1	12 (17.91%)	15 (23.44%)		
	- T2	22 (32.83%)	20 (31.25%)		
	- T3	18 (26.87%)	17 (26.56%)		
	- T4	15 (22.39%)	12 (18.75%)		
TNM stage (N)				3.164	0.367
	- N0	25 (37.31%)	28 (43.75%)		
	- N1	18 (26.87%)	19 (29.69%)		
	- N2	15 (22.39%)	14 (21.88%)		
	- N3	9 (13.43%)	3 (4.68%)		
Baseline ECOG performance status				1.729	0.421
	- 0	31 (46.27%)	35 (54.69%)		
	- 1	25 (37.31%)	23 (35.94%)		
	- 2	11 (16.42%)	6 (9.38%)		

NPC, nasopharyngeal carcinoma; MBSR, mindfulness-based stress reduction; TNM, tumor node metastasis; ECOG, Eastern Cooperative Oncology Group.

### Psychological Parameters Before Treatment and After 8 Weeks of Treatment

As shown in Table 4, there was no significant difference in HAMD-17, SDS, SF-12, PSSS, and MAAS scores between the control group and the MBSR treatment group before treatment (*p* > 0.05). However, after treatment, compared to the control group, the MBSR treatment group demonstrated a significant decrease in HAMD-17 and SDS scores, along with a substantial increase in SF-12, PSSS, and MAAS scores (*p* < 0.05). These findings suggest that MBSR has a positive effect in treating moderate depression induced by NPC.

**Table 4.** **Comparison of psychological parameters before treatment and after 8 weeks of treatment**.

Parameters		Control group (n = 67)	MBSR therapy group (n = 64)	*t*-value	*p*-value
HAMD-17	Before treatment	19.63 ± 1.87	19.52 ± 1.86	0.342	0.733
	After treatment	15.84 ± 2.16	10.64 ± 2.47	12.834	<0.001
SDS	Before treatment	66.54 ± 3.25	66.88 ± 3.27	–0.593	0.554
	After treatment	62.66 ± 2.32	56.33 ± 2.92	13.760	<0.001
PSSS	Before treatment	22.48 ± 3.45	23.16 ± 3.18	1.175	0.242
	After treatment	31.48 ± 3.67	33.12 ± 3.84	2.495	0.014
SF-12	Before treatment	53.48 ± 2.67	52.79 ± 2.91	1.416	0.159
	After treatment	54.73 ± 4.34	56.29 ± 4.37	2.050	0.042
MAAS	Before treatment	28.75 ± 3.47	27.89 ± 3.59	1.394	0.166
	After treatment	32.15 ± 7.21	35.06 ± 7.58	2.251	0.026

MBSR, mindfulness-based stress reduction; HAMD-17, Hamilton Depression Scale-17; SDS, Self-Rating Depression Scale; SF-12, Short-From-12 Health Survey; PSSS, Perceived Social Support Scale; MAAS, Mindful Attention Awareness Scale.

### Patients’ Satisfaction

The assessment of patient satisfaction revealed statistically significant differences between the control and MBSR groups. As shown in Table 5, these differences were found in overall satisfaction with treatment (t = –2.030, *p* = 0.044), willingness to recommend the treatment (χ^2^ = 5.378, *p* = 0.020), and perceived benefit from the therapy (χ^2^ = 7.141, *p* = 0.008). These results indicate a higher level of satisfaction, increased willingness to recommend the treatment, and greater perceived benefit from MBSR therapy among patients with NPC-induced moderate depression, emphasizing its positive impact on patient experience and outcomes.

**Table 5.** **Patients’ satisfaction with treatment**.

Parameters	Control group (n = 67)	MBSR therapy group (n = 64)	*t*/χ^2^	*p*-value
Overall satisfaction with treatment	6.74 ± 1.48	7.26 ± 1.47	–2.030	0.044
Willingness to recommend treatment	49 (73.13%)	57 (89.06%)	5.378	0.020
Perceived benefit from treatment	34 (50.75%)	47 (73.44%)	7.141	0.008

MBSR, mindfulness-based stress reduction.

## Discussion

NPC poses significant challenges to patients, affecting them physically and psychologically [24]. The disease and its treatments can lead to various psychological issues, including depression, anxiety, and reduced quality of life [25, 26]. Consequently, there was a growing interest in identifying adjunctive therapeutic approaches to address the psychological well-being of patients with NPC-induced moderate depression [27]. Therefore, we evaluated the adjunctive therapeutic potential of MBSR therapy for patients diagnosed with NPC and moderate depression.

MBSR equips individuals with self-regulation skills that can be used in daily life to manage stress, pain, and illness. Among diverse cancer patients, MBSR has positively impacted clinical symptoms and emotional well-being [28, 29]. In this study, we observed that compared to the control group, NPC patients who underwent 8 weeks of MBSR treatment demonstrated significant improvement in depression levels, aligning with previous findings [30]. These observations indicate that MBSR treatment may positively impact the mental health of NPC patients. Due to the high mortality rate of cancer, patients often experience strong fear and adverse reactions during radiotherapy and chemotherapy [31], rendering them more prone to depression, anxiety, and other negative emotions. MBSR training guides patients to accept themselves, acknowledge their current emotions, experiences, or behaviors, and learn to manage their negative emotions. This approach aims to prevent patients from falling into negative emotions, thereby enabling them to maintain a calm and rational attitude in facing various treatment process challenges.

Our study also revealed that, compared to the control group, the MBSR treatment group significantly improved perceived stress, quality of life, and mindfulness attention awareness. Furthermore, MBSR training improves patients’ abilities to manage stress by elevating their mindfulness attention awareness, enabling them to focus on the current movement and reduce interruptions from external disturbances. The improvement in psychological parameters after MBSR treatment aligns with the principle of mindfulness, which emphasizes current-focused attention, non-judgment, and acceptance of their experience [32]. These principles can enhance psychological resilience, help manage painful symptoms, and foster emotional regulation for NPC patients.

Furthermore, the MBSR treatment group tailored training methods based on the cognitive characteristics and depression levels of NPC patients, incorporating patient-centered care principle and emphasizing the importance of addressing the unique needs of individuals with complex medical and psychological conditions [33, 34]. This study also evaluated patient satisfaction, demonstrating that patients in the MBSR treatment group reported higher satisfaction and a stronger willingness to recommend this treatment. These findings highlight the positive impact of MBSR treatment on patient experience and treatment outcomes, indicating that this intervention has been appreciated and valued by patients. The emphasis on patient satisfaction and perceived benefits is consistent with the broader goal of patient-centered care, which prioritizes meeting patient needs, preferences, and experiences in the provision of health care services.

While this study demonstrates that MBSR therapy provides promising adjunctive therapeutic value for patients with NPC-induced moderate depression, several limitations should be noted. Firstly, the retrospective study design limits the ability to establish causal relationships between MBSR therapy and the observed improvements in psychological outcomes. Future prospective studies, particularly randomized controlled trials, could provide stronger evidence of the causal impact of MBSR therapy on psychological well-being in this patient group. Additionally, the study’s focus on a specific patient population—individuals with NPC-induced moderate depression—may limit the generalizability of the findings to other patient groups with different medical and psychological conditions.

## Conclusion

In summary, the findings of this study support the adjunctive therapeutic value of MBSR therapy in improving psychological outcomes and patient satisfaction among individuals with NPC-induced moderate depression. The patient-centered approach, tailored training methods, and ethical considerations underscore the potential of MBSR therapy as a valuable addition to the comprehensive care for NPC patients. Further research, including prospective studies and considerations of contextual factors, is warranted to advance our understanding of the broader implications and clinical utility of MBSR therapy for this patient group.

## Availability of Data and Materials

Data to support the findings of this study are available on reasonable request from the corresponding author.
